# Special Issue “Genomics of Fungal Plant Pathogens”

**DOI:** 10.3390/jof9070713

**Published:** 2023-06-29

**Authors:** Baohua Wang, Yakubu Saddeeq Abubakar, Zonghua Wang

**Affiliations:** 1 State Key Laboratory of Ecological Pest Control for Fujian and Taiwan Crops, Ministerial and Provincial Joint Innovation Centre for Safety Production of Cross-Trait Crops, College of Plant Protection, Fujian Agriculture and Forestry University, Fuzhou 350002, China; wbaohua@fafu.edu.cn (B.W.); ay.saddeeq@yahoo.com (Y.S.A.); 2 Department of Biochemistry, Faculty of Life Science, Ahmadu Bello University, Zaria 810281, Nigeria; 3 Institute of Oceanography, Minjiang University, Fuzhou 350108, China

Plant diseases can be classified according to pathogenic organisms, and 70–80% of them are fungal diseases. Fungal diseases cause a significant reduction in crop yield and quality and threaten global food security. More than 20,000 species of fungi are known to cause diseases in crops and plants. As of 5 June 2023, the genome data of over 4239 different fungal species have been published and are available at the NCBI database (https://www.ncbi.nlm.nih.gov/genome/browse#!/overview/, accessed on 5 June 2023). Comparative genomes of fungal plant pathogens provided great insight into fungal genomic compositions and structures.

*Pyricularia oryzae* (syn. *Magnaporthe oryzae*), *Botrytis cinerea* and *Fusarium graminearum* are ranked first, second and fourth of the top ten global fungal plant pathogens [1]. These fungal species are now considered to be model systems for the study of plant–fungus pathogen interactions. In this Special Issue, 156 genome assemblies were used to construct a pan-genome of *P. oryzae*; then, the mechanisms of genetic divergence and virulence variation of different sub-populations were elucidated. The pan-genome contained a total of 24,100 genes—twice that of the reference genome 70-15 strain, including 70% of core genes and 5% of strain-specific genes. Conventional secreted proteins were enriched in high-frequency near-core genes and localized proximate to transposable elements (TEs). The virulence genes *AVR1-CO39*, *AVR-Pi54*, *AvrPi9* and *AvrPiz-t* are present in all of the investigated isolates, except for *AvrPii* and *Avr-Pia* [2].

Moreover, the genome sequence data of six other fungal plant pathogens were performed, and their genome sizes ranged from 38.25 Mb to 66 Mb (Table 1). These data provided new insights into the genome sizes and the composition of fungal plant pathogens and laid a solid foundation for further studies on the mechanisms of pathogenicity, on morphological characteristics, on forward/population genetics, on molecular markers, and on disease management.

Ultimately, one of the direct ways to determine how a gene (and the protein it encodes) functions in cellular processes is to see what happens to the organism when that gene is lacking or overexpressed. Genes are contained within the nucleus, mitochondria and chloroplasts (plant) in eukaryotes. In this Special Issue, the function of two mitochondrial genes in *Botrytis cinerea* and *Fusarium graminearum* were elucidated. One is mitochondrial transport protein (MTP), which catalyzes the transport of biochemical substances across the mitochondrial inner membrane. Shao et al. [7] generated the *Bcmtp1* mutant of *B. cinerea*. The results demonstrate that BcMTP1 is involved in the regulation of the vegetative growth, asexual reproduction, stress tolerance and virulence of *B. cinerea*. Han et al. [8] investigated *F. graminearum* mitrochondrial porin, or voltage-dependent anion-selective channels, which regulate the complex interactions associated with organellar and cellular metabolism. The authors generated *Fgporin* mutant in *F. graminearum* and characterized the function of FgPorin. The results showed that FgPorin is involved in the regulation of fungal hyphal growth, conidiation, sexual reproduction, virulence on wheat and autophagy.

The small Rho GTPase family regulates most fundamental processes of eukaryotic cells, including (but not limited to) morphogenesis, polarity, movement, cell division, gene expression and cytoskeleton reorganization [9,10]. In order to elucidate the function of the small GPTase MoRho3 in *M. oryzae*, Li et al. [11] performed comparative transcriptomic analysis of *MoRho3* constitutively active mutant (*MoRho3-CA*) and *MoRho3* dominant-negative mutant (*MoRho3-DN*). In *MoRho3-CA* vs. WT, about 874 up-regulated, differentially expressed genes (DEGs) were detected, and the DEGs were significantly enriched in the ribosome biogenesis pathway, while 1511 down-regulated DEGs were also detected and were enriched in different amino acids and chemical metabolism pathways. Meanwhile, in *MoRho3-DN* vs. WT, the authors detected 986 up-regulated DEGs which were enriched in genes associated with some metabolic pathways, ABC transporters and regulators for autophagy. They similarly detected 1215 down-regulated DEGs which were enriched in genes associated with some selected metabolic pathways. This reveals that MoRho3 plays crucial roles in ribosome biogenesis and protein secretions. In another study, Zheng et al. [12] used another phytopathogenic fungus, *Fusarium odoratissimum*, to elucidate the function of a small GTPase FoSec4. The results also showed that FoSec4 plays a crucial role in vegetative growth, reproduction, pathogenicity and response to environmental stress in *F. odoratissimum*.

Sorting nexins (SNX) are a highly conserved and diverse family of cellular trafficking proteins that confer a wide variety of functions, including signal transduction, membrane deformation and cargo binding. Moreover, sorting nexins are key modulators of endosome dynamics and autophagic functions [13]. In this Special Issue, Yu et al. [14] generated *Chsnx4* and *Chsnx41* mutants and elucidated their functions in *Cochliobolus heterostrophus*. The results demonstrated that both *ChSNX4* and *ChSNX41* are involved in regulating vegetative growth, asexual reproduction, appressorium formation, oxidative stress, adaptation to antifungal agents and virulence of *C. heterostrophus*. These phenotypes are similar to those characterized in *M. oryzae* and *F. graminearum* [15,16,17,18,19]. This indicates that, at the very least, the morphological functions of *SNX4* and *SNX41* are similar in phytopathogenic fungi.

Members of the Glycosyltransferase 2 (GT2) family include cellulose synthase, chitin synthase, glycosyl-transferase, mannosyltransferase, galactosyltransferase, rhamnosyltransferase, etc. [20]. Glycosyltransferases (GT) play crucial roles in fungal biosynthesis pathways, including fungal cell wall synthesis, and many glycosyltransferases are unique to these fungi [21]. In this Special Issue, Blandenet et al. [22] generated the membrane protein glycosyl-transferase BcCps1 deletion mutant in *B. cinerea* and elucidated the functions of BcCps1. The results indicate that BcCps1 is essential for the mycelial growth, sexual reproduction, stress tolerance and cell wall biosynthesis of *B. cinerea*. These phenotypes are also similar to those observed in *M. oryzae*, *F. graminearum*, *F. verticillioides* and *Zymoseptoria tritici* [23,24,25], indicating that the morphological functions of Cps1 are similar in phytopathogenic fungi.

The regulator family of protein, zinc binuclear cluster proteins (Zn(II)2Cys6), are unique to fungi. Zn(II)2Cys6 play crucial roles in fungal development, carbon and nitrogen utilizations, secondary metabolites biosynthesis, stress response, virulence, chromatin remodeling and so on. In this Special Issue, Bansal et al. [26] used the Zn(II)2Cys6 coding sequences from nine ascomycetes phytopathogenic fungal species and yeast to analyze their composition and codon usage bias patterns. The nine fungal species were divided into two major groups based on their *zinc binuclear cluster* coding sequences, and the phytopathogenic fungal species in cluster-1 (*B. maydis*, *B. oryzae*, *Alternaria alternate*, *F. graminearum* and *Aspergillus flavus*) showed a lower number of GC-rich high-frequency codons than the species in cluster-2 (*Gaeumannomyces tritici*, *P. oryzae*, *Colletotrichum graminicola* and *Verticillium dahliae*), while *C. cerevisiae* tends to be AT-rich. The presence of Zn(II)2Cys6 GC-rich codons could facilitate the invasion process. The results also showed that specific codons and sequences can modulate the interaction between a host and pathogen through genome editing functional genomics tools.

In plant pathology, unveiling the mechanisms of host–pathogen interactions is of paramount importance. From both sides, many genes are involved in the process. In this Special Issue, Wang et al. [27] obtained 229 isolates of *Blumeria graminis* (*Bgh*) and analyzed their virulence and genetic traits. Isolates form Yunnan showed the highest diversity in virulence complexity and genetic diversity. The results demonstrated that inter-group genetic variation was 54.68%, while inter- and intra-group genetic variation were 21.4% and 23.9%, respectively. The results indicated that the *Bgh* population in Tibet has undergone expansion recently, resulting in increased virulence on wheat and a loss of genetic diversity. These results are similar to the virulence and genetic diversity of *B. graminis* in Southeastern and Southwestern China [28].

In conclusion, we would like to thank all authors who contributed to this Special Issue on “Genomics of Fungal Plant Pathogens”. We also thank each of the peer reviewers who provided valuable comments on the 13 manuscripts, and the members of the *Journal of Fungi* Editorial Office, for their support.

## Figures and Tables

**Table 1:** Genome assembly features of six fungal plant pathogens.

Pathogen	Genome Size (Mb)	Predicted Genes	GC Content	Repeat Sequence	Reference
*Calonectria ilicola*	66.3	18,366	48%	1.53%	[3]
*Diaporthe amygdali*	51.5	15,818	52.1%	1.1109%	[4]
*Diaporthe eres*	60.8	16,499	47.6%	1.3151%	[4]
*Bipolaris zeicola*	32.21	10,108	50.66%	11.63%	[5]
*Exserohilum* *rostratum*	34.05	10,457	50.56%	3.7%	[5]
*Stagonospora tainanensis*	38.25	12,206	51.49%	13.20%	[6]

## References

[1] Dean R., Van Kan J.A.L., Pretorius Z.A., Hammond-Kosack K.E., Di Pietro A., Spanu P.D., Rudd J.J., Dickman M., Kahmann R., Ellis J. (2012). The Top 10 fungal pathogens in molecular plant pathology. Mol. Plant Pathol..

[2] Bao J., Wang Z., Chen M., Chen S., Chen X., Xie J., Tang W., Zheng H., Wang Z. (2022). Pan-genomics reveals a new variation pattern of secreted proteins in *Pyricularia oryzae*. J. Fungi.

[3] Chen X., Luo M., Wu W., Dong Z., Zou H. (2022). Virulence-associated genes of *Calonectria ilicola*,responsible for *Cylindrocladium* black rot. J. Fungi.

[4] Hilário S., Gonçalves M.F.M., Fidalgo C., Tacão M., Alves A.J. (2022). Genome analyses of two blueberry pathogens: *Diaporthe amygdali* CAA958 and *Diaporthe eres* CBS 160.32. J. Fungi.

[5] He K., Zhao C., Zhang M., Li J., Zhang Q., Wu X., Wei S., Wang Y., Chen X., Li C. (2023). The chromosome-scale genomes of *Exserohilum rostratum* and *Bipolaris zeicola* pathogenic fungi causing rice spikelet rot disease. J. Fungi.

[6] Xu F., Li X., Ren H., Zeng R., Wang Z., Hu H., Bao J., Que Y.J. (2022). The first telomere-to-telomere chromosome-level genome assembly of *Stagonospora tainanensis* causing sugarcane leaf blight. J. Fungi.

[7] Shao W., Zhang Y., Chen C., Xing Y. (2023). Function of the mitochondrial transport protein BcMtp1 in regulating vegetative development, asexual reproduction, stress response, fungicide sensitivity, and virulence of *Botrytis cinerea*. J. Fungi.

[8] Han X., Li Q., Li X., Lv X., Zhang L., Zou S., Yu J., Dong H., Chen L., Liang Y. (2022). Mitochondrial porin is involved in development, virulence, and autophagy in *Fusarium graminearum*. J. Fungi.

[9] Mosaddeghzadeh N., Ahmadian M.R. (2021). The RHO Family GTPases: Mechanisms of Regulation and Signaling. Cells.

[10] Jaffe A.B., Hall A. (2005). Rho GTPases biochemistry and Biology. Annu. Rev. Cell Dev. Biol..

[11] Li Q., Chen X., Lin L., Zhang L., Wang L., Bao J., Zhang D. (2022). Transcriptomic dynamics of active and inactive states of Rho GTPase MoRho3 in *Magnaporthe oryzae*. J. Fungi.

[12] Zheng Y., Guo P., Deng H., Lin Y., Huang G., Wu J., Lu S., Yang S., Zhou J., Zheng W. (2022). Small GTPase FoSec4-mediated protein secretion is important for polarized growth, reproduction and pathogenicity in the banana Fusarium wilt fungus *Fusarium odoratissimum*. J. Fungi.

[13] Hanley S.E., Cooper K.F. (2020). Sorting Nexins in Protein Homeostasis. Cells.

[14] Yu H., Jia W., Li Z., Gao C., Pan H., Zhang X. (2022). The sorting nexin genes *ChSNX4* and *ChSNX41* are required for reproductive development, stress adaption and virulence in *Cochliobolus heterostrophus*. J. Fungi.

[15] Deng Y.Z., Qu Z., He Y., Naqvi N.I. (2012). Sorting nexin Snx41 is essential for conidiation and mediates glutathione-based antioxidant defense during invasive growth in *Magnaporthe oryzae*. Autophagy.

[16] Deng Y., Qu Z., Naqvi N.I. (2013). The role of Snx41-based pexophagy in *Magnaporthe* development. PLoS ONE.

[17] He Y., Deng Y.Z., Naqvi N.I. (2013). Atg24-assisted mitophagy in the foot cells is necessary for proper asexual differentiation in *Magnaporthe oryzae*. Autophagy.

[18] Lv W., Xu Z., Talbot N.J., Wang Z. (2020). The sorting nexin FgAtg20 is involved in the Cvt pathway, non-selective macroautophagy, pexophagy and pathogenesis in *Fusarium graminearum*. Cell Microbiol..

[19] Zheng W., Lin Y., Fang W., Zhao X., Lou Y., Wang G., Zheng H., Liang Q., Abubakar Y.S., Olsson S. (2018). The endosomal recycling of FgSnc1 by FgSnx41-FgSnx4 heterodimer is essential for polarized growth and pathogenicity in *Fusarium graminearum*. New Phytol..

[20] Breton C., Šnajdrová L., Jeanneau C., Koča J., Imberty A. (2006). Structures and mechanisms of glycosyltransferases. Glycobiology.

[21] Klutts J.S., Yoneda A., Reilly M.C., Bose I., Doering T.L. (2006). Glycosyltransferases and their products: *Cryptococcal* variations on fungal themes. FEMS Yeast Res..

[22] Blandenet M., Gonçalves I.R., Rascle C., Dupuy J.-W., Gillet F.-X., Poussereau N., Choquer M., Bruel C. (2022). Evidencing new roles for the glycosyl-transferase Cps1 in the phytopathogenic fungus *Botrytis cinerea*. J. Fungi.

[23] Deng S., Sun W., Dong L., Cui G., Deng Y.Z. (2019). *MoGT2* is essential for morphogenesis and pathogenicity of *Magnaporthe oryzae*. Msphere.

[24] Deng Q., Wu H., Gu Q., Tang G., Liu W. (2021). Glycosyltransferase FvCpsA regulates fumonisin biosynthesis and virulence in *Fusarium verticillioides*. Toxins.

[25] King R., Urban M., Lauder R.P., Hawkins N., Evans M., Plummer A., Halsey K., Lovegrove A., Hammond-Kosack K., Rudd J.J. (2017). A conserved fungal glycosyltransferase facilitates pathogenesis of plants by enabling hyphal growth on solid surfaces. PLoS Pathog..

[26] Bansal S., Mallikarjuna M.G., Balamurugan A., Nayaka S.C., Prakash G. (2022). Composition and codon usage pattern results in divergence of the *zinc binuclear cluster* (*Zn(II)_2_Cys_6_*) sequences among Ascomycetes plant pathogenic fungi. J. Fungi.

[27] Wang Y., Zhuoma Q., Xu Z., Peng Y., Wang M. (2023). Virulence and genetic types of *Blumeria graminis* f. sp. *hordei* in Tibet and surroundingareas. J. Fungi.

[28] Wang Y., Zhang G., Wang F., Lang X., Zhao X., Zhu J., Hu C., Hu J., Zhang Y., Yao X. (2023). Virulence variability and genetic diversity in *Blumeria graminis* f. sp. *hordei* in Southeastern and Southwestern China. Plant Dis..

